# 5-(Thio­phen-2-ylmeth­yl)-1,3,4-thia­diazol-2-amine

**DOI:** 10.1107/S1600536812013633

**Published:** 2012-04-04

**Authors:** Yavuz Köysal, Sadık Deniz, Ray J. Butcher, Sema Öztürk Yildirim, Jerry P. Jasinski, Amanda C. Keeley

**Affiliations:** a Yeşilyurt Demir Çelik Vocational School, Ondokuz Mayıs University, Samsun, Turkey; b Department of Chemistry, Karadeniz Technical Universty, 61080 Trabzon, Turkey; c Department of Chemistry, Howard University, 525 College Street, NW, Washington, DC 2059, USA; d Department of Physics, Faculty of Sciences, Erciyes University, 38039 Kayseri, Turkey; e Department of Chemistry, Keene State College, 220 Main Street, Keene, NH 03435-2001, USA

## Abstract

In the title mol­ecule, C_7_H_7_N_3_S_2_, the dihedral angle between the thio­phene and thia­diazole rings is 72.99 (5)°; the two rings are oriented so that the S atoms in each ring are on the same side. In the crystal, the three-dimensional network involves strong N—H⋯O hydrogen bonds, as well as C—H⋯π and π–π stacking inter­actions [centroid–centroid distances = 3.654 (1) and 3.495 (1) Å].

## Related literature

For the anti­tumor activity of 2-amino-1,3,4-thia­diazole, 2-ethyl­amino-1,3,4-thia­diazole and 2,2′-(methyl­enediamino)­bis-1,3,4-thia­diazole, see: Olesan *et al.* (1955 ▶); Mishra *et al.* (1995 ▶). For their anti-HIV, anti­proliferative, germicidal and D2 dopamine­rgic activity, see: Mohareb *et al.* (2004 ▶). For the synthesis of the title compound, see: Sancak *et al.*, (2007 ▶). For standard bond lengths, see: Allen *et al.* (1987 ▶).
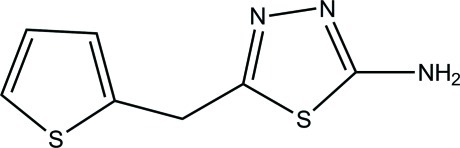


## Experimental

### 

#### Crystal data

C_7_H_7_N_3_S_2_
*M* *_r_* = 197.28
Monoclinic, 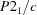

*a* = 11.2970 (6) Å
*b* = 6.6094 (3) Å
*c* = 11.2480 (6) Å
β = 97.243 (5)°
*V* = 833.15 (7) Å^3^
*Z* = 4
Cu *K*α radiation
μ = 5.33 mm^−1^
*T* = 173 K
0.46 × 0.28 × 0.15 mm

#### Data collection

Agilent Xcalibur Eos Gemini diffractometer
Absorption correction: multi-scan (*CrysAlis PRO*; Agilent, 2010 ▶) *T*_min_ = 0.209, *T*_max_ = 0.450
4375 measured reflections
1539 independent reflections
1497 reflections with *I* > 2σ(*I*)
*R*_int_ = 0.036

#### Refinement

*R*[*F* ^2^ > 2σ(*F* ^2^)] = 0.045
*wR*(*F* ^2^) = 0.120
*S* = 1.09
1539 reflections
110 parameters
H-atom parameters constrained
Δρ_max_ = 0.64 e Å^−3^
Δρ_min_ = −0.38 e Å^−3^

### 

Data collection: *CrysAlis PRO* (Agilent, 2010 ▶); cell refinement: *CrysAlis PRO*; data reduction: *CrysAlis PRO*; program(s) used to solve structure: *SHELXS97* (Sheldrick, 2008 ▶); program(s) used to refine structure: *SHELXL97* (Sheldrick, 2008 ▶); molecular graphics: *SHELXTL* (Sheldrick, 2008 ▶); software used to prepare material for publication: *SHELXTL*.

## Supplementary Material

Crystal structure: contains datablock(s) I, global. DOI: 10.1107/S1600536812013633/hg5202sup1.cif

Structure factors: contains datablock(s) I. DOI: 10.1107/S1600536812013633/hg5202Isup2.hkl

Supplementary material file. DOI: 10.1107/S1600536812013633/hg5202Isup3.cml

Additional supplementary materials:  crystallographic information; 3D view; checkCIF report

## Figures and Tables

**Table 1:** Hydrogen-bond geometry (Å, °) *Cg* is the centroid of the S1/C1–C4 ring.

*D*—H⋯*A*	*D*—H	H⋯*A*	*D*⋯*A*	*D*—H⋯*A*
N3—H3*B*⋯N1^i^	0.86	2.13	2.991 (2)	175
N3—H3*A*⋯N2^ii^	0.86	2.17	3.013 (2)	167
C1—H1⋯*Cg*^iii^	0.93	2.83	3.549 (2)	135

Symmetry codes: (i) 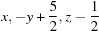; (ii) 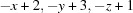; (iii) 
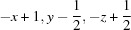
.

**Fig. 1.**
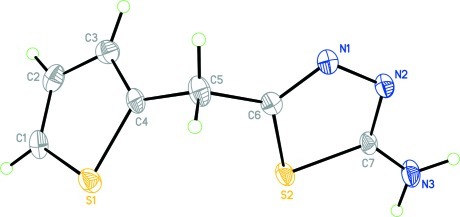
View of the molecular structure of the title compound with the atom-numbering scheme. Displacement ellipsoids are drawn at the 50% probability level for non-hydrogen atoms.

**Fig. 2.**
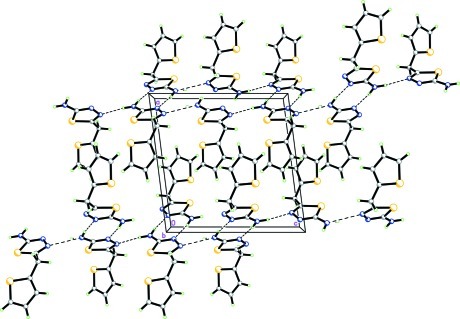
The packing view showing the hydrogen bonds network. Dashed lines indicate intermolecular N—H···N hydrogen bonds (see Table 1 for details).

## supplementary crystallographic information

### Comment

The antitumor activites of 2-amino-1,3,4-thiadiazole (ATDA, NSC-4728) and the related compounds: 2-ethylamino-1,3,4-thiadiazole (EATDA), 2,2'-(methylene-diamino) bis-1,3,4-thiadiazole (NSC-143019) were found in several experimental tumor systems about 50 years ago (Olesan *et al.*, 1955). 2-Amino-1,3,4-thiadiazole (ATDA), as the most promising compound, was used in phase II clinical trials in patients with different tumors: renal, colon, ovarian, and others. Recently new derivatives with the 1,3,4-thiadiazole nucleus as well as Fe(II) / Fe (III) complexes of 2-amino-1,3,4-thiadiazoles have been synthesized and evaluated for their antiproliferative activity against a panel of human cancer cell lines (Mishra *et al.*, 1995). Over recent years, there has been an increasing interest in the chemistry of thiophenes because of their biological significance. Many of them have been widely investigated for therapeutic uses, especially as antifungal, antibacterial, anti-inflammatory, anticonvulsant, antiasthmatic, and analgesic agents. They also were known to show anti-HIV, antiproliferative, germicidal, and D2 dopaminergic activities (Mohareb *et al.*, 2004). In view of these facts, the aim of this present study is to obtain a structure of 1,3,4-oxadiazole incorporating the thiophene ring.

In the molecule of the title compound (Fig 1), the bond lengths are within normal ranges (Allen *et al.*, 1987). In the molecule of (I) atom S1 is oriented towards the thiadiazol ring, Fig. 1. The dihedral angle between the planar thiophene (r.m.s. deviation = 0.007 Å) and planar thiadiazol (r.m.s. deviation = 0.004 Å) rings of 72.99 (5)° indicates a twist between planes as seen in the S1–C4–C5–C6 torsion angle of 94.86 (17) °. The amine group is effectively co-planar with the thiadiazol ring to which it is attached as seen in the N3–C7–S2–C6 torsion angle of 178.53 (16) °.

In the crystal structure, there are strong intermolecular N–H···N hydrogen bonds which lead to the formation of centrosymmetric dimers in the crystal. In addition there are C—H···π and π-π stacking interactions [*Cg*1···*Cg*1(1 - *x*, -*y*, -*z*) = 3.654 (1) Å and *Cg*2···*Cg*2(-*x*, 1 - *y*, -*z*) = 3.495 (1) Å, *Cg*1(S1/C1—C4) and *Cg*2(S2/N1/N2/C6/C7) are the centroids of the thiophene and thiadiazol rings]. This pattern is the primary supramolecular structure for this compound (Fig. 2).

### Experimental

The title compound was synthesized using the published method (Sancak *et al.*, 2007).

### Refinement

The amine H atoms were seen in a difference Fourier map and then idealized with *U*_iso_(H) = 1.2*U*_eq_(N) with N–H bond length of 0.86 Å. The C-bound H-atoms were positioned geometrically with C—H = 0.93 and 0.97 Å, for aromatic and CH_2_ H-atoms, respectively, and constrained to ride on their parent atoms, with *U*_iso_(H) = 1.2*U*_eq_(C). During the refinement it was noticed that for the strongest reflections (F_c_/F_c_(max) close to 1.00) the observed value (F_o_) was much smaller than the calculated value (F_c_) indicating detector saturation problems. These reflections were omitted from the refinement.

### Crystal data

**Table:**

C_7_H_7_N_3_S_2_	*F*(000) = 408
*M**_r_* = 197.28	*D*_x_ = 1.573 Mg m^−^^3^
Monoclinic, *P*2_1_/*c*	Cu *K*α radiation, λ = 1.54184 Å
Hall symbol: -P 2ybc	Cell parameters from 2744 reflections
*a* = 11.2970 (6) Å	θ = 3.9–70.0°
*b* = 6.6094 (3) Å	µ = 5.33 mm^−^^1^
*c* = 11.2480 (6) Å	*T* = 173 K
β = 97.243 (5)°	Chunk, colorless
*V* = 833.15 (7) Å^3^	0.46 × 0.28 × 0.15 mm
*Z* = 4	

### Data collection

**Table:**

Agilent Xcalibur Eos Gemini diffractometer	1539 independent reflections
Radiation source: Enhance (Cu) X-ray Source	1497 reflections with *I* > 2σ(*I*)
Graphite monochromator	*R*_int_ = 0.036
Detector resolution: 16.1500 pixels mm^-1^	θ_max_ = 70.1°, θ_min_ = 3.9°
ω scans	*h* = −13→13
Absorption correction: multi-scan (*CrysAlis PRO*; Agilent, 2010)	*k* = −5→8
*T*_min_ = 0.209, *T*_max_ = 0.450	*l* = −12→13
4375 measured reflections	

### Refinement

**Table:**

Refinement on *F*^2^	Secondary atom site location: difference Fourier map
Least-squares matrix: full	Hydrogen site location: inferred from neighbouring sites
*R*[*F*^2^ > 2σ(*F*^2^)] = 0.045	H-atom parameters constrained
*wR*(*F*^2^) = 0.120	*w* = 1/[σ^2^(*F*_o_^2^) + (0.0928*P*)^2^ + 0.0849*P*] where *P* = (*F*_o_^2^ + 2*F*_c_^2^)/3
*S* = 1.09	(Δ/σ)_max_ = 0.001
1539 reflections	Δρ_max_ = 0.64 e Å^−^^3^
110 parameters	Δρ_min_ = −0.38 e Å^−^^3^
0 restraints	Extinction correction: *SHELXL97* (Sheldrick, 2008), Fc^*^=kFc[1+0.001xFc^2^λ^3^/sin(2θ)]^-1/4^
Primary atom site location: structure-invariant direct methods	Extinction coefficient: 0.035 (3)

### Special details

**Table:**

Geometry. All e.s.d.'s (except the e.s.d. in the dihedral angle between two l.s. planes) are estimated using the full covariance matrix. The cell e.s.d.'s are taken into account individually in the estimation of e.s.d.'s in distances, angles and torsion angles; correlations between e.s.d.'s in cell parameters are only used when they are defined by crystal symmetry. An approximate (isotropic) treatment of cell e.s.d.'s is used for estimating e.s.d.'s involving l.s. planes.
Refinement. Refinement of *F*^2^ against ALL reflections. The weighted *R*-factor *wR* and goodness of fit *S* are based on *F*^2^, conventional *R*-factors *R* are based on *F*, with *F* set to zero for negative *F*^2^. The threshold expression of *F*^2^ > σ(*F*^2^) is used only for calculating *R*-factors(gt) *etc*. and is not relevant to the choice of reflections for refinement. *R*-factors based on *F*^2^ are statistically about twice as large as those based on *F*, and *R*- factors based on ALL data will be even larger.

### Fractional atomic coordinates and isotropic or equivalent isotropic displacement parameters (Å^2^)

**Table:**

	*x*	*y*	*z*	*U*_iso_*/*U*_eq_	
S1	0.64166 (4)	0.57641 (7)	0.32224 (4)	0.0210 (2)	
S2	0.82410 (4)	1.05521 (6)	0.34150 (3)	0.0182 (2)	
N1	0.87197 (12)	1.0981 (2)	0.56747 (13)	0.0175 (4)	
N2	0.91662 (12)	1.2714 (2)	0.51908 (13)	0.0179 (4)	
N3	0.93579 (14)	1.4168 (2)	0.33358 (14)	0.0251 (4)	
H3A	0.9732	1.5198	0.3665	0.030*	
H3B	0.9220	1.4085	0.2568	0.030*	
C1	0.48911 (16)	0.5889 (3)	0.30609 (17)	0.0216 (4)	
H1	0.4395	0.5306	0.2430	0.026*	
C2	0.44958 (15)	0.6943 (3)	0.39653 (16)	0.0226 (4)	
H2	0.3693	0.7160	0.4030	0.027*	
C3	0.54422 (16)	0.7680 (3)	0.48045 (16)	0.0206 (4)	
H3	0.5323	0.8420	0.5483	0.025*	
C4	0.65455 (15)	0.7192 (2)	0.45147 (15)	0.0171 (4)	
C5	0.77380 (16)	0.7709 (3)	0.51957 (16)	0.0212 (4)	
H5A	0.8306	0.6668	0.5044	0.025*	
H5B	0.7667	0.7689	0.6046	0.025*	
C6	0.82264 (14)	0.9732 (3)	0.48849 (14)	0.0159 (4)	
C7	0.89906 (14)	1.2698 (3)	0.40174 (15)	0.0165 (4)	

### Atomic displacement parameters (Å^2^)

**Table:**

	*U*^11^	*U*^22^	*U*^33^	*U*^12^	*U*^13^	*U*^23^
S1	0.0161 (3)	0.0215 (4)	0.0250 (4)	−0.00005 (14)	0.0010 (2)	−0.00692 (15)
S2	0.0184 (3)	0.0192 (3)	0.0158 (3)	−0.00404 (13)	−0.0027 (2)	−0.00264 (13)
N1	0.0172 (7)	0.0156 (7)	0.0191 (7)	−0.0010 (5)	0.0000 (6)	0.0004 (5)
N2	0.0170 (7)	0.0175 (8)	0.0184 (7)	−0.0032 (5)	−0.0012 (5)	−0.0010 (5)
N3	0.0272 (9)	0.0290 (9)	0.0172 (8)	−0.0138 (6)	−0.0039 (6)	0.0017 (6)
C1	0.0172 (8)	0.0152 (8)	0.0311 (9)	−0.0020 (6)	−0.0017 (7)	0.0002 (7)
C2	0.0176 (8)	0.0169 (9)	0.0337 (10)	0.0024 (6)	0.0052 (7)	0.0078 (7)
C3	0.0248 (9)	0.0135 (9)	0.0239 (9)	0.0041 (6)	0.0054 (7)	0.0012 (6)
C4	0.0211 (8)	0.0084 (8)	0.0213 (9)	−0.0011 (6)	0.0007 (7)	0.0000 (6)
C5	0.0236 (9)	0.0130 (9)	0.0251 (9)	−0.0007 (6)	−0.0039 (7)	0.0016 (7)
C6	0.0139 (7)	0.0152 (8)	0.0179 (8)	0.0022 (6)	−0.0004 (6)	−0.0003 (6)
C7	0.0100 (7)	0.0184 (9)	0.0199 (8)	−0.0002 (6)	−0.0025 (6)	−0.0031 (6)

### Geometric parameters (Å, º)

**Table:**

S1—C1	1.7119 (18)	C1—C2	1.354 (3)
S1—C4	1.7237 (17)	C1—H1	0.9300
S2—C6	1.7419 (17)	C2—C3	1.420 (3)
S2—C7	1.7445 (17)	C2—H2	0.9300
N1—C6	1.287 (2)	C3—C4	1.366 (2)
N1—N2	1.390 (2)	C3—H3	0.9300
N2—C7	1.310 (2)	C4—C5	1.503 (2)
N3—C7	1.336 (2)	C5—C6	1.505 (2)
N3—H3A	0.8600	C5—H5A	0.9700
N3—H3B	0.8600	C5—H5B	0.9700
			
C1—S1—C4	92.29 (9)	C3—C4—C5	127.66 (16)
C6—S2—C7	86.93 (8)	C3—C4—S1	110.36 (13)
C6—N1—N2	113.90 (14)	C5—C4—S1	121.96 (13)
C7—N2—N1	111.83 (14)	C4—C5—C6	114.53 (14)
C7—N3—H3A	120.0	C4—C5—H5A	108.6
C7—N3—H3B	120.0	C6—C5—H5A	108.6
H3A—N3—H3B	120.0	C4—C5—H5B	108.6
C2—C1—S1	111.59 (14)	C6—C5—H5B	108.6
C2—C1—H1	124.2	H5A—C5—H5B	107.6
S1—C1—H1	124.2	N1—C6—C5	123.32 (15)
C1—C2—C3	112.57 (16)	N1—C6—S2	113.61 (13)
C1—C2—H2	123.7	C5—C6—S2	123.00 (12)
C3—C2—H2	123.7	N2—C7—N3	123.65 (16)
C4—C3—C2	113.16 (16)	N2—C7—S2	113.71 (13)
C4—C3—H3	123.4	N3—C7—S2	122.63 (13)
C2—C3—H3	123.4		
			
C6—N1—N2—C7	−0.27 (19)	N2—N1—C6—C5	176.73 (15)
C4—S1—C1—C2	−1.08 (14)	N2—N1—C6—S2	−0.36 (18)
S1—C1—C2—C3	0.4 (2)	C4—C5—C6—N1	136.56 (17)
C1—C2—C3—C4	0.7 (2)	C4—C5—C6—S2	−46.6 (2)
C2—C3—C4—C5	−179.68 (15)	C7—S2—C6—N1	0.64 (13)
C2—C3—C4—S1	−1.50 (19)	C7—S2—C6—C5	−176.45 (15)
C1—S1—C4—C3	1.47 (14)	N1—N2—C7—N3	−178.55 (16)
C1—S1—C4—C5	179.78 (14)	N1—N2—C7—S2	0.78 (18)
C3—C4—C5—C6	−87.1 (2)	C6—S2—C7—N2	−0.80 (13)
S1—C4—C5—C6	94.86 (17)	C6—S2—C7—N3	178.53 (16)

### Hydrogen-bond geometry (Å, º)

Cg is the centroid of the S1/C1–C4 ring.

**Table:**

*D*—H···*A*	*D*—H	H···*A*	*D*···*A*	*D*—H···*A*
N3—H3*B*···N1^i^	0.86	2.13	2.991 (2)	175
N3—H3*A*···N2^ii^	0.86	2.17	3.013 (2)	167
C1—H1···*Cg*^iii^	0.93	2.83	3.549 (2)	135

Symmetry codes: (i) *x*, −*y*+5/2, *z*−1/2; (ii) −*x*+2, −*y*+3, −*z*+1; (iii) −*x*+1, *y*−1/2, −*z*+1/2.
